# Outcomes, Symptomatology, and Mortality of Children Presenting With Bacterial Meningitis at Allied Hospitals of Rawalpindi Medical University, Pakistan: A Cross-Sectional Study

**DOI:** 10.7759/cureus.56107

**Published:** 2024-03-13

**Authors:** Muhammad Zarak Khan, Aiman Waheed, Faizan Fazal, Shahrukh Ahmad Khan, Ehsan Ahmad, Sanan Rasheed, Talha Ijaz, Areesha Abid, Saima Ambreen, Bilal Haider Malik

**Affiliations:** 1 Department of Medicine, Holy Family Hospital, Rawalpindi, PAK; 2 Internal Medicine, California Institute of Behavioral Neurosciences & Psychology, Fairfield, USA

**Keywords:** bacterial meningitis, pediatric mortality, outcome analysis, pediatric infectious disease, sign and symptoms

## Abstract

Introduction

Bacterial meningitis (BM) is a neurologic emergency mainly affecting children under the age of two. Clinical symptoms are rarely evident in children, thus making a diagnosis is a challenge. Antibiotic therapy should be started timely to ensure the avoidance of significant morbidity and mortality. This study aims to assess the outcomes, mortality, and symptomatology of children presenting with BM in allied hospitals of Rawalpindi Medical University, Pakistan.

Methods

It is a cross-sectional study employing a sample size of 201, conducted at the Allied Hospitals of Rawalpindi Medical University, Pakistan from a period of January 2023 to August 2023. Non-probability convenience sampling was used. Children aged between newborns and 14 years of age with a confirmed diagnosis of bacterial meningitis were included in this study. The study population was divided into five different age groups. Three different outcomes were studied including complete recovery, development of complications, and death. Data was entered into and analyzed by Statistical Package for the Social Sciences (SPSS) version 25 (IBM Corp., Armonk, NY, USA). Descriptive statistics were applied to the demographic data. The chi-square analytical test was applied to study the association between the categorical variables.

Results

One hundred nineteen (59.2%) of the study’s population were males. One hundred twenty-six (62.7%) of the patients were born through a spontaneous vaginal delivery (SVD). The majority (54%) of the study population were infants. Twenty-three percent were newborns, 13% were toddlers, 6% were preschool children, and 4% were school-age children. The majority (85%) of the study participants belonged to lower socioeconomic backgrounds. Ninety percent of the cases had symptoms of fever, seizures, and poor feeding. Neck stiffness was significantly associated with death as an outcome (p-value=0.01). The overall mortality amongst the study population was 20%. Forty-nine percent of the study population recovered completely, whereas 31% had complications following the diagnosis. Neonates had a higher mortality rate than infants (45% vs 9% respectively).

Conclusion

The most common presenting symptoms were fever, vomiting, seizures, and neck stiffness. Poor feeding was also seen in most cases. The rate of complications and death is observed to be relatively higher following the diagnosis of bacterial meningitis as compared to rates in the surrounding and developed countries. Out of all signs and symptoms, the presence of neck stiffness was significantly associated with death as an outcome among children with bacterial meningitis.

## Introduction

Children under the age of two are most frequently afflicted by bacterial meningitis (BM), a potentially fatal inflammation of the meninges [1]. BM is a neurologic emergency requiring immediate attention. The prevalence of the disease has decreased because of vaccination against common pathogens. Early diagnosis and prompt administration of adjunctive and empiric antibiotic therapy are essential. Before any imaging tests, therapy should start as soon as blood cultures have been collected [1]. Fever, headache, meningismus, and altered level of consciousness are clinical indications that suggest BM; however, they may be rare in children, the elderly, and people with meningococcal illness [2].

Children older than newborns are among those most at risk for contracting BM [3]. In the past, Streptococcus pneumoniae, Neisseria meningitidis, and Haemophilus influenzae serotype B (Hib) were the main pathogens responsible for infecting this population globally [3]. An epidemiological study on Tunisian children concluded that in terms of etiology, pneumococcus was predominantly responsible (69.84%) followed by meningococcus (28.57%) and Hib (1.59%) [4]. In children, clinical diagnosis is challenging to evaluate. Additionally, standard bacteriological methods have a limited sensitivity for instances where they have already received antibiotic treatment and cerebrospinal fluid (CSF) samples are of poor quality. Cases of BM are still underreported, which emphasizes the need for high-performance detection techniques like real-time polymerase chain reaction (PCR) [5]. Since the first conjugate vaccines against the three bacteria were developed, the epidemiology of BM has evolved. In fact, in affluent nations, 90% fewer occurrences of Hib meningitis were reported after receiving the Hib vaccine [6].

A study conducted in South Africa across 26 hospitals concluded that HIV infection, comorbidities, and altered mental status were risk factors for death as an outcome in patients with BM antimicrobial nonsusceptibility and impaired mental status were risk factors for negative sequelae [7]. A 50-year study in Sweden on the etiology, risk factors, disease trends, and severe sequelae concluded that following the introduction of the vaccination, the BM incidence in immunosuppressed individuals climbed by 3% yearly. The 30-day death rate was 3% for infants and 14% for adults, and 44% of patients experienced serious sequelae. Patients lost an average of 11 healthy years of life as a result of BM [8]. A study aimed at observing the neurological sequelae showed that in 27% of neurological sequelae, there were hearing abnormalities in 15%, cognitive impairment in 12%, and motor or sensory nerve deficits in 9%. Three percent of patients died, while 18% of patients had unfavorable outcomes [9]. Thus, it is essential to study and assess the epidemiology related to BM. It is also important to study mortality rates among different age groups in the pediatric population. This study aims to assess the outcomes, mortality, and symptomatology of children presenting with BM in allied hospitals of Rawalpindi Medical University, Pakistan.

## Materials and methods

Study design, duration, and setting

This is a cross-sectional study by design. It was conducted from January 2023 to August 2023 at the Allied Hospitals of Rawalpindi Medical University, Pakistan including Holy Family Hospital and Benazir Bhutto Hospital.

Study population, sample size, and sampling technique

The study population was the children with ages ranging from birth to 14 years. The sample size of this study was 201 with a population proportion of 24.5% calculated by the World Health Organization sample size calculator. The sampling technique used in this study was nonprobability convenience sampling.

Eligibility criteria

The eligibility criteria were defined before the initiation of the study. Children with confirmed bacterial meningitis were included in the study. Children with viral, fungal, or aseptic meningitis and children whose parents did not grant consent were excluded from this study.

Variables included in the study

The main variables observed in this study included the demographic variables and the outcome variables. The demographic data included details related to gender, economic status, and mode of delivery. The study population was divided into five different age groups comprising newborns (0 days to one month), infants (one month to one year), toddlers (one year to three years), preschool children (three years to six years), and school-age children (six years to 14 years). Three different outcomes were studied: complete recovery, development of complications, and death.

Data analysis

Data was entered into and analyzed by Statistical Package for the Social Sciences (SPSS) version 25 (IBM Corp., Armonk, NY, USA). Descriptive statistics were applied to the demographic data. The chi-square test was applied to study the association between the categorical variables. A P-value of less than 0.05 was considered significant.

Ethical approval and budget

Before the initiation of this study, ethical approval was taken from the ethical review board of Rawalpindi Medical University. The reference number of the approval letter is M-33-46-22. It is a self-financed study; no funding was taken from any source.

## Results

Demographics of the study population

59.2% (n=119) of the study’s population were males whereas 40.8% (n=82) were females. 62.7% (n=126) of the patients were born through spontaneous vaginal delivery (SVD). 37.3% (n=75) were born through a Cesarean section. The demographic details are provided in Table 1.

**Table 1:** Demographics of the study population

Variables	Frequency n=201	Percentage (%)
Gender		
Male	119	59.2%
Female	82	40.8%
Economic status		
High socioeconomic status	7	3.4%
Middle socioeconomic status	24	12%
Low socioeconomic status	170	84.6%
Mode of delivery		
Cesarean section	75	37.3%
Spontaneous vaginal delivery (SVD)	126	62.7%

Outcomes of patients with bacterial meningitis

**Figure 1:**
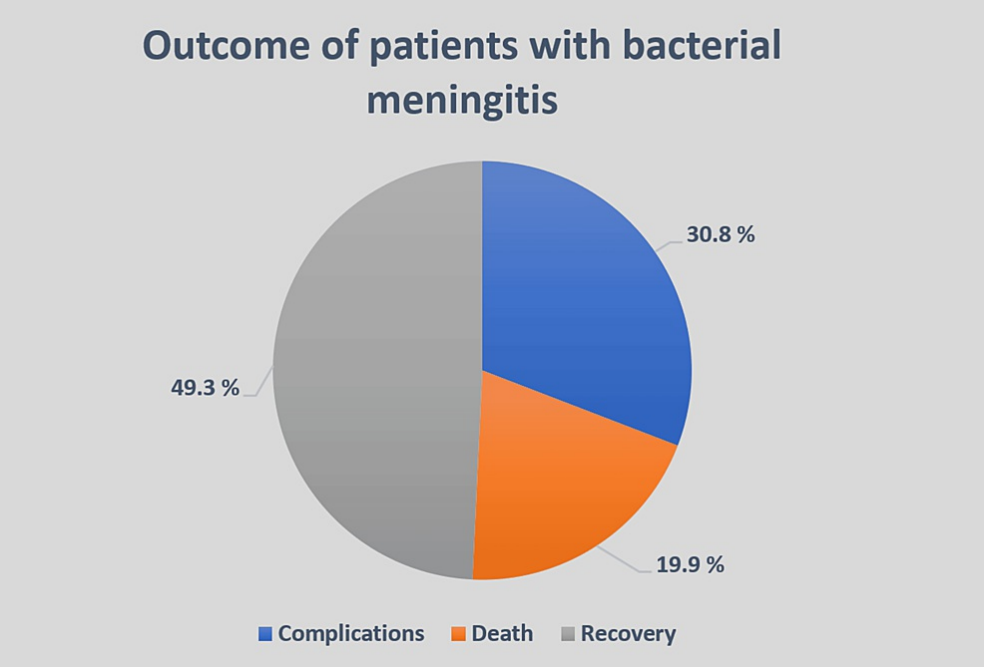
Outcomes of patients with bacterial meningitis

49.3% (n=99) of the patients recovered completely without any complications. 19.9% (n=40) of the patients ultimately died due to bacterial meningitis, whereas 30.8% (n=62) of the patients had developed complications following bacterial meningitis. This is shown in Figure 1.

Prevalence of various symptoms in bacterial meningitis

Fever was present in 92% of the cases. Seizures were observed in 90% of the patients. Neck stiffness was present in 48% of the cases. The rest of the details are given in Table 2.

**Table 2:** Various symptoms in patients of bacterial meningitis

Symptoms	Present N(%)	Absent N(%)
Fever	185 (92%)	16 (8%)
Vomiting	88 (43.7%)	113 (56.3%)
Neck stiffness	97 (48.2%)	104 (51.8%)
Seizures	182 (90.5%)	19 (9.5%)
Poor Feeding	183 (91%)	18 (9%)

Signs and symptoms of bacterial meningitis and their association with outcome

The presence of neck stiffness among children with bacterial meningitis was found to be significantly associated with death as an outcome (p-value=0.01). The association of other signs and symptoms and their association with the outcome are shown in Table 3.

**Table 3:** Signs and symptoms of bacterial meningitis and their association with the outcome

Symptoms	Present N (%)	Absent N (%)	Chi-square value	P-value
Fever	185 (92%)	16 (8%)	2.04	0.36
Neck stiffness	97 (48%)	104 (52%)	8.68	0.01
Poor feeding	183 (91%)	18 (9%)	5.21	0.26
Seizures	182 (90%)	19 (10%)	8.51	0.07
Vomiting	87 (43%)	114 (57%)	1.90	0.75
Brudzinski's sign	117 (58%)	84 (42%)	1.62	0.44
Kernig's sign	118 (58%)	83 (42%)	1.75	0.41

Mortality rates in different age groups

Thirty-nine (19.4%) out of 201 patients ultimately died from bacterial meningitis. Neonates had the highest mortality amongst all age groups. Forty-five percent of the neonates died from bacterial meningitis. Infants had the lowest rate of mortality. Only 9% of the infants died from bacterial meningitis. This is shown in Table 4.

**Table 4:** Mortality rates in different age groups

	Mortality	Percentage	Chi-square value	p-value
Newborn	21/46	45%	26.28	0.000
Infant	10/108	9%	15.35	0.000
Toddler	3/26	11%	1.18	0.28
Pre-school	2/12	16%	0.06	0.80
School-age child	3/9	33%	1.16	0.28

## Discussion

The vast majority of the parents of this study’s participants belong to lower socioeconomic backgrounds. This would be expected in any developing country. The socioeconomic factor alone is an independent risk factor for the occurrence of community-acquired BM [6]. It is associated with increased susceptibility to invasive bacterial infections in both children and adults [10]. This study also shows that the majority of the study’s participants were born through SVD. It has been shown that pathogens that have colonized the mother's vagina are one of the causes of early-onset BM in neonates [11]. This study shows that around 60% of the participants were males. Another study on the etiology of bacterial meningitis conducted in Nairobi, Kenya showed that 54% of the patients in their study were males.

Approximately half of the study’s participants eventually recovered after being treated for BM. Twenty percent died whereas others had complications following bacterial meningitis. This outcome measure is crucial for further planning by healthcare staff so that the number of patients being recovered can be increased and necessary interventions can decrease complications. A study conducted in Seoul, South Korea on 161 cases of childhood BM showed that mortality was 15% [12]. A study across three continents showed that the mortality among 2123 children with BM was 3%, 13%, and 38% in Finland, LatAm, and Angola, respectively [13]. This study also shows that fever, seizures, and poor feeding were present in more than 90% of the cases of BM. Vomiting and neck stiffness were present in less than 50% of the cases. A study on predictive signs and symptoms of BM conducted in Ghana showed that the most frequently reported signs and symptoms of probable BM were fever, stiff neck, headache, convulsions, and altered consciousness [14]. This study has further shown that the presence of neck stiffness among children with BM is significantly associated with death as an outcome. Thus, the patients presenting with neck stiffness as one of the main complaints should be considered as a priority case. This study also observed the statistics related to mortality observed in each age group of the study population. The highest mortality due to BM was observed in the neonatal age group. The lowest mortality was observed in the infant age group. Because their humoral and cellular immune systems are still developing, neonates have a higher chance of developing meningitis. In addition, because they lack distinct clinical symptoms, diagnosing meningitis in newborns is more challenging than in older kids. Pediatricians continue to face a public health problem in the form of neonatal meningitis. Depending on the term of diagnosis, the type of discovered organisms, and the time passed before receiving therapy, mortality and long-term sequelae are seen in 10-15% and 20-50% of survivors, respectively [15].

The vast majority of the participants in the study were from lower socioeconomic groups. Fever, vomiting, seizures, and stiff neck were the most frequent presenting symptoms. In most cases, poor feeding was also evident. The rate of complications and death is relatively higher following the diagnosis of BM compared to rates in the surrounding and developed countries. The occurrence of neck stiffness was strongly related to death as a result in children with BM, out of all the signs and symptoms. One of the limitations of this study is the use of convenient sampling. The data has been gathered from one major district of Pakistan, it would have been better to get data from a number of various districts to have a better understanding of the whole country's population. Nevertheless, the data included and gathered for this study is unique in the sense that this type of study has not been performed in recent years in Pakistan. Thus, this study provided unique insights into the patients diagnosed with BM.

## Conclusions

The vast majority of the study population belonged to lower socioeconomic status. The most common presenting symptoms were fever, vomiting, seizures, and neck stiffness. Poor feeding was also seen in most cases. The rate of complications and death is observed to be relatively higher following the diagnosis of bacterial meningitis as compared to rates in the surrounding and developed countries. Out of all signs and symptoms, the presence of neck stiffness was significantly associated with death as an outcome among children with bacterial meningitis.
